# Self-reported patient history to assess hepatitis B virus serological status during a large screening campaign

**DOI:** 10.1017/S0950268818002650

**Published:** 2018-09-28

**Authors:** A. Boyd, J. Gozlan, F. Carrat, H. Rougier, P.-M. Girard, K. Lacombe, J. Bottero

**Affiliations:** 1 INSERM, UMR_S1136, Institut Pierre Louis d'Epidémiologie et de Santé Publique, F-75012, Paris, France; 2 Laboratoire de Virologie, Hôpital Saint-Antoine, AP-HP, F-75012, Paris, France; 3 INSERM, UMR_S1135 CIMI, F-75013, Paris, France; 4 Département de santé publique, Hôpital Saint-Antoine, AP-HP, F-75012, Paris, France; 5 Sorbonne Universités, INSERM, UPMC Univ Paris 06, Institut Pierre Louis d’épidémiologie et de Santé Publique (IPLESP UMRS 1136), F-75012, Paris, France; 6 Service de maladies infectieuses, Hôpital Saint-Antoine, AP-HP, F-75012, Paris, France

**Keywords:** Disease status, infection awareness, serology, vaccination, validation

## Abstract

When assessing hepatitis B virus (HBV) status in clinical settings, it is unclear whether self-reports on vaccination history and previous HBV-test results have any diagnostic capacity. Of 3997 participants in a multi-centre HBV-screening study in Paris, France, 1090 were asked questions on their last HBV-test result and vaccination history. Discordance between self-reported history compared with infection status (determined by serology) was calculated for participants claiming ‘negative’, ‘effective vaccine’, ‘past infection’, or ‘chronic infection’ HBV-status. Serological testing revealed that 320 (29.4%) were non-immunised, 576 (52.8%) were vaccinated, 173 (15.9%) had resolved the infection and 21 (1.9%) were hepatitis B surface antigen positive. In total 208/426 (48.8%) participants with a self-reported history of ‘negative’ infection had a discordant serological result, in whom 128 (61.5%) were vaccinated and 74 (35.6%) had resolved infections. A total of 153/599 (25.5%) participants self-reporting ‘effective vaccine’ had a discordant serological result, in whom 100 (65.4%) were non-immunised and 50 (32.7%) were resolved infections. Discordance for declaring ‘past’ or ‘chronic infection’ occurred in 9/55 (16.4%) and 3/10 (30.0%) individuals, respectively. In conclusion, self-reported HBV-status based on participant history is partially inadequate for determining serological HBV-status, especially between negative/vaccinated individuals. More adapted patient education about HBV-status might be helpful for certain key populations.

## Introduction

Approximately 18.5 million persons are infected with hepatitis B virus (HBV) in Europe [1]. Epidemiological evidence suggests that roughly three-quarters of them are unaware of their infection [2], which, if left poorly managed or untreated during active phases, could lead to further progression of liver disease, hepatocellular carcinoma and death [3, 4]. Vaccine coverage rates are also low in many countries across Europe [5], leaving a considerable proportion of individuals at risk of acquiring HBV. Consequently, there is an urgent need to identify infected persons requiring appropriate care and to determine those eligible for HBV vaccination.

Several HBV screening guidelines have been proposed and implemented over recent years and recommend an approach targeting populations at-risk of infection [6]. Routine screening has been largely successful in identifying infected individuals at sexually-transmitted disease (STD) clinics and among immigrant populations from moderate to high HBV-endemic regions, sex-workers and intravenous-drug users (IDUs) [7–9]. Furthermore, testing has been simplified with the development of rapid point-of-care technology, which has provided a quick and convenient means to accurately test for infection and link patients to care [10].

Nevertheless, in France, there were a total of 43 18 664 tests given for 33 962 HBsAg-positive cases in 2016 [11] and a large number of these tests could be regarded as unnecessary [7]. Among individuals already tested for HBV, it is uncertain whether repeat screening could provide any public health benefit, especially if the individual is considered no longer at risk of infection [12, 13]. HBV testing could be reduced by basing the need to test on patient history, yet this would depend on the reliability of patient recollection. For example, self-reports of vaccinated status have demonstrated poor concordance compared with serological results in IDUs [14, 15], individuals testing in STD clinics [16] and men who have sex with men (MSM) [17]. In one study conducted in Paris, France, a false understanding of vaccinated status was more frequently observed in individuals with previous HBV testing [18]. Reported vaccination status alone is unlikely to assist in determining testing eligibility.

Combining patients’ understanding of their previous HBV test with HBV vaccination history could help more accurately assess patients’ HBV status and thus need to test. However, very few studies have evaluated this approach. In the present study, we aimed to explore the validity of individual self-reported HBV-status obtained from questions related to participants’ history (i.e. vaccinated status and previous HBV test result) compared with serological results during a mass screening campaign in Paris, France.

## Methods

### Study design and participants

The OPTISCREEN-B program is a multicentre cross-sectional study, whose primary objective was to evaluate several rapid tests for chronic HBV [10]. Briefly, ten healthcare centres were selected to represent those whose objectives included screening, prevention and/or vaccination of diverse populations. More specifically, these centres provided one of the following services: free and anonymous sexually transmitted disease (STD) testing (*Consultation de dépistage anonyme et gratuite* (CDAG) Belleville, CDAG Figuier, CDAG St Antoine); testing for the general population (*Centre d'examens de santé de la Caisse Primaire d'Assurance Maladie* (CPAM), *Consultation Voyage St Antoine*), immigrants and persons with low socio-economic status (*Centre de Santé rue au Maire*, *Médecins du Monde*, *Policlinique St Antoine*, *Croix-Rouge Moulin Joly*), or incarcerated individuals (*Unité de consultations et de soins ambulatoires* (UCSA)). All centres were required to be in the Paris metropolitan region so that HBV-infected patients could be referred to a single hospital (Saint-Antoine Hospital, Paris) for subsequent care.

The target screening population was persons potentially eligible for HBV-testing. Since one major factor steering the decision to test in practice is complete certainty of prior HBV-infection or vaccination status, participants with a confirmed HBsAg-positive, anti-HBsAg antibody-positive, or anti-HBcAg antibody-positive test (requiring proof of result) were not included. This means that participants were still eligible if they declared having a previous test or vaccination but were unable to provide proof. Participants whose result was negative for all three HBV serological markers >6 months prior were also eligible. Importantly, no participant was excluded based on nationality, legal situation or access to government-provided healthcare.

From September 2010 to August 2011, volunteers were asked to participate during their regular consultation if ⩾18-years-old and available for further contact. For the present study, only individuals claiming previous HBV testing were included. All participants provided written informed consent in their native language and the protocol was approved by the Hôtel-Dieu Hospital Ethics Committee (Paris, France) in accordance with the Helsinki Declaration.

### HBV risk-factor questionnaire

A questionnaire was administered by a trained clinical research assistant during a face-to-face interview. Questions were asked in lay terms on a variety of socio-demographic characteristics, healthcare coverage and HBV transmission risk-factors. HBV-endemicity of the birth country was established according to WHO classification (prevalence of HBsAg-positive individuals): high (>8%), intermediate (2–8%) and low (<2%).

### HBV serological battery and definition of HBV-disease status

Full screening procedures have been detailed elsewhere [10]. A complete HBV serological battery was performed for all participants and included HBsAg, anti-HBs antibodies, hepatitis B ‘e’ antigen (HBeAg), anti-hepatitis B ‘e’ (HBe) antibodies and anti-HBc antibodies (MONOLISA AgHBs Ultra, anti-HBs plus, anti-hepatitis B core antibody-anti-HBc-plus, BIORAD, Hercules, USA). For this study, HBV-disease status was classified using serological results as follows: non-immunised, resolved infection, vaccinated and HBsAg-positive (Table 1).

**Table 1.** Definition of HBV infection status

HBV infection status	HBV serology		
	HBsAg	anti-HBc Ab	anti-HBs Ab
Non-immunised	–	–	–
Vaccinated	–	–	+
Resolved infection^a^	–	+	+/−
HBsAg-positive	+	+/−	+/−

Ab, antibodies; HBc, hepatitis B core; HBs, hepatitis B surface; HBsAg, HBs antigen; HBV, hepatitis B virus.

a Individuals with isolated anti-HBc antibody positive serology (*n* = 37) were regrouped with those having resolved infection in the analysis.

### Assessing HBV-related participant history

Participants were asked whether they had a previous HBV-test and could answer ‘yes’, ‘no’ or ‘do not know or remember’. Individuals responding ‘yes’ were then questioned on whether they obtained a test result and if so, which of the following best described their result: ‘negative (no past exposure)’, ‘meant that your vaccine worked’, ‘meant that your infection was cured’, ‘meant that you have chronic infection’, or ‘do not know or remember’. Tested participants were also asked to specify the reason for testing, choosing one of the following: ‘as part of a normal health check-up’, ‘during blood donation’, ‘before getting vaccinated for hepatitis B’, ‘problems from liver tests’, or ‘because I have a transmission risk-factor’.

Participants were also asked whether they had been vaccinated against HBV infection and could answer ‘yes’, ‘no’ or ‘do not know or remember’. Individuals responding ‘yes’ were then questioned on the age at which they were vaccinated (either continuous or specified as during ‘birth’, ‘adolescence’, or ‘adulthood’), the number of vaccinations they received and whether they verified protective antibodies with a serological test any time after vaccination.

### Statistical analysis

Based on participants’ answers to questions on vaccination history and previous HBV-test result, we constructed four self-reported HBV-status groups: (i) negative, (ii) effective vaccine, (iii) past infection and (iv) chronic infection. Any individual who claimed HBV vaccination prior to participation (responding ‘yes’ to the question on HBV vaccination) was classified as belonging to the ‘effective vaccine’ group. All other individuals were then categorised in groups corresponding to their previous HBV-test result. We compared participant self-reported *vs.* serological HBV-infection status using unweighted *κ*.

Using serological testing as the gold standard, discordance was defined as the conditional probability of not having a true disease status given the participant's self-report (1-sensitivity). Risk-factors for discordance were evaluated for each self-report group, provided that there were a sufficient number of participants (*n* > 100). Unadjusted odds ratios (OR) and their 95% confidence intervals (CI) were calculated from random-effects logistic regression models accounting for within-centre correlation (due to centre-specific experience with healthcare services) [19].

All statistical analyses were performed using STATA (v12.1, College Station, TX, USA) statistical software and significance was determined using a *P*-value <0.05.

## Results

### Description of the study population

A total of 3997 individuals participated in the initial HBV screening study. Of them, 2907 were not included because they had incomplete or unavailable serological results (*n* = 68), did not have a previous HBV-test (*n* = 2120), were unsure of having an HBV test (*n* = 610), or did not obtain their last HBV-test result (*n* = 109). In total, 1090 participants were included in the analysis. Included individuals were more likely to have a risk factor for HBV transmission or to come from a country of intermediate/high HBV prevalence compared with those non-included (Supplementary Table 1).

Table 2 describes patient characteristics in function of self-reported HBV-status. Participants reporting past infection and the effective vaccine had significantly older and younger median ages, respectively, when compared with other groups (*P* < 0.001). As expected, many of the factors related to regions of moderate-to-high HBV-endemicity were significantly more frequent in participants declaring past or chronic infection (Table 2). Other significant differences in health insurance plan, sexual activity and previous incarceration were also observed between self-reported HBV-status groups.

**Table 2.** Characteristics of the study population

	Self-report from participant's history^a^				*P*^b^
	Negative	Effective vaccine	Past infection	Chronic infection	
	(*n* = 426)	(*n* = 599)	(*n* = 55)	(*n* = 10)	
Male	251 (58.9)	351 (58.6)	40 (72.7)	5 (50.0)	0.2
Age^c^ (*N* = 1089)	32 (25–42)	32 (25–40)	45 (35–52)	38 (26–45)	<0.001
HBV prevalence of birth country					<0.001
Low (<2.0%)	253 (59.4)	429 (71.6)	12 (21.8)	0 (0)	
Intermediate (2.0–8.0%)	101 (23.7)	78 (13.0)	36 (65.5)	9 (90.0)	
High (>8.0%)	72 (16.9)	92 (15.4)	7 (12.7)	1 (10.0)	
Parents born in high HBV-endemic region (*N* = 1087)	123 (28.9)	125 (21.0)	36 (65.5)	9 (90.0)	<0.001
Travelled to high HBV-endemic region^d^	123 (28.9)	118 (19.7)	36 (65.5)	9 (90.0)	<0.001
Sought care in high HBV-endemic region	91 (21.4)	74 (12.4)	28 (50.9)	9 (90.0)	<0.001
Health insurance plan					<0.001
Social security	342 (80.3)	538 (89.8)	34 (61.8)	5 (50.0)	
CMU^e^	20 (4.7)	22 (3.7)	2 (3.6)	1 (10.0)	
AME^f^	25 (5.9)	9 (1.5)	5 (9.1)	1 (10.0)	
Other	4 (0.9)	1 (0.2)	1 (1.8)	0 (0)	
None	35 (8.2)	29 (4.8)	13 (23.6)	3 (30.0)	
Received transfusion before 1992	11 (2.6)	22 (3.7)	3 (5.5)	1 (10.0)	0.4
Received acupuncture	63 (14.8)	97 (16.2)	7 (12.7)	2 (20.0)	0.8
Received tattoos	71 (16.7)	93 (15.5)	9 (16.4)	1 (10.0)	0.9
Received piercing	190 (44.6)	279 (46.6)	16 (29.1)	3 (30.0)	0.07
Close contact with an HBV + individual	40 (9.4)	71 (11.9)	6 (10.9)	5 (50.0)	0.001
Number of life-time sexual partners					<0.001
0–1	34 (8.0)	29 (4.8)	9 (16.4)	3 (30.0)	
2–9	179 (42.0)	226 (37.7)	18 (32.7)	5 (50.0)	
⩾10	213 (50.0)	344 (57.4)	28 (50.9)	2 (20.0)	
>1 sexual partner within the last 12 months	233 (54.7)	390 (65.1)	23 (41.8)	4 (40.0)	<0.001
Men who have sex with men	60 (14.1)	177 (29.6)	10 (18.2)	0 (0)	<0.001
Nasal drug-use	69 (16.2)	105 (17.5)	6 (10.9)	0 (0)	0.3
Intravenous drug-use	4 (0.9)	11 (1.8)	1 (1.8)	0 (0)	0.7
Long-term stay at a medical centre	21 (4.9)	19 (3.2)	3 (5.5)	0 (0)	0.4
Previously incarcerated	29 (6.8)	22 (3.7)	7 (12.7)	1 (10.0)	0.01

Data from the Optiscreen-B study conducted from September 2010 to August 2011 in Paris, France among individuals reporting having been tested for hepatitis B virus (HBV).

a Self-report according to accounts of participant's vaccination history and previous test.

b Overall comparisons between self-report groups were performed using Kruskal–Wallis test for continuous variables and Pearson *χ*^2^ test for categorical variables.

c Median (IQR) given.

d Period of stay was longer than 3 months.

e *Couverture médicale universelle*, health insurance coverage that is given to persons living in precarious situations (i.e. unemployed, poverty, etc.).

f *Aide médicale d’état*, health insurace generally given to immigrants without proper documentation.

### Previous HBV testing and vaccination

Participants reported having the following results from a previous HBV-test: negative, *n* = 835 (76.6%); effective vaccination, *n* = 181 (16.6%); past infection, *n* = 59 (5.4%); and chronic infection, *n* = 15 (1.4%). No one reported not knowing or remembering their test result. Previous tests were conducted as part of routine care (*n* = 586, 53.8%) or because the participant had an HBV-transmission risk-factor (*n* = 455, 41.7%), were about to receive HBV-vaccination (*n* = 21, 1.9%), donate blood (*n* = 14, 1.3%), or had abnormal liver enzymes or symptoms of liver-related illness (*n* = 14, 1.3%). Of those testing due to an HBV-transmission risk factor (*n* = 453 with available data), 397 (86.6%) had recent sexual contact, 15 (3.3%) had close contact with a chronically-infected individual, 14 (3.1%) were required to test for HBV by their employer, nine (2.0%) were from a region of high HBV-prevalence, six (1.3%) had occupational exposure to HBV, five (1.1%) were an IDU, 4 (0.9%) had a transfusion prior to 1992, 2 (0.4%) were travelling to a high HBV-endemic country, and 6 (1.3%) had miscellaneous risk-factors.

A total of 595 (54.6%) participants claimed to be previously vaccinated and 217 (19.9%) did not know or remember whether they were vaccinated. Among those certain of being vaccinated, vaccinations occurred mostly during adulthood (54.6%) and less commonly during adolescence (34.5%) or childhood/birth (10.9%). Of the 357 individuals who were able to recall the number of vaccinations administered, 52.9% had three or more vaccinations, 28.9% two and 18.2% only one. Almost half of the vaccinated participants (*n* = 283/595, 47.6%) stated that they verified protection with a serological test.

Of the 835 individuals reporting that their last HBV-test was negative, 409 (49.0%) claimed to receive HBV vaccination during their lifetime. No inference on the sequence of testing and vaccination can be made as these data were not collected. Of the 181 individuals with a past HBV-test reported as ‘effective vaccine’, 177 (97.8%) stated that they were vaccinated for HBV.

### Differences in self-report *vs.* serological HBV-status

During screening, serological testing revealed that 320 (29.4%) were non-immunised, 576 (52.8%) were vaccinated (with median anti-HBs antibody level at 451 mIU/ml, IQR = 76->1000), 173 (15.9%) had resolved infection (with median anti-HBs antibody level at 137 mIU/ml, IQR = 14–929) and 21 (1.9%) were HBsAg-positive. In Table 3, self-reported history of HBV-disease status is compared with serological results. There were 717 (65.8%) participants whose self-report agreed with the study test, giving an unweighted *κ* = 0.42. Of note, an agreement based on only self-reported test results from the previous HBV-test was much poorer (Supplementary Table 2). When examining individual disease status groups (with self-report/serology, respectively), an agreement was similar between negative/non-immunised (*κ* = 0.37), effective vaccine/vaccinated (*κ* = 0.48), past infection/resolved infection (*κ* = 0.35) and chronic infection/HBsAg-positive (*κ* = 0.44).

**Table 3.** Self-reported HBV-infection status compared with serological results

Serological HBV-status^a^	Self-reported status^b^			
	Negative	Effective vaccine	Past infection	Chronic infection
	(*n* = 426)	(*n* = 599)	(*n* = 55)	(*n* = 10)
Non-immunised	218 (51.2)	100 (16.7)	2 (3.6)	0 (0)
Vaccinated	128 (30.1)	446 (74.5)	2 (3.6)	0 (0)
Resolved infection	74 (17.4)	50 (8.4)	46 (83.6)	3 (30.0)
HBsAg-positive	6 (1.4)	3 (0.5)	5 (9.1)	7 (70.0)

Data from the Optiscreen-B study conducted from September 2010 to August 2011 in Paris, France among individuals reporting having been tested for hepatitis B virus (HBV).

a Disease status was defined according to serological results (given in Table 1).

b Self-report was based on responses to the following questions: ‘Have you already been vaccinated against hepatitis B?’ and ‘What was the result of [your last] HBV test?’ Status was defined as follows: any individual who claimed HBV vaccination prior to participation was classified as belonging to the ‘effective vaccine’ group; all other individuals were then categorised in groups corresponding to their previous HBV-test result.

### Discordance in HBV-status and its determinants

Of the 426 participants with negative self-reported history, 208 (48.8%) had a discordant serological result. Of them, 74 (35.6%) were resolved infections (with median anti-HBs antibody level at 96 mIU/ml, IQR = 10–425), 128 (61.5%) vaccinated (with median antibody level at 439 mIU/ml, IQR = 52->1000) and six (2.9%) had HBsAg-positive serology. As shown in Table 4, higher odds of negative HBV-infection discordance were found in individuals from an intermediate endemic country (*P* = 0.04), had parents from, travelled to, or received care in a region of intermediate/high HBV-prevalence (*P* < 0.001) or who had CMU as their health insurance. Lower odds were observed in those from a high HBV-prevalent country (*P* = 0.02) and with more than one-lifetime sexual partner (*P* = 0.01) (Table 4). No multivariable analysis was conducted due to strong collinearity between variables, notably region of endemicity.

**Table 4.** Determinants for discordant hepatitis B virus status

	Negative (*n* = 426)		Effective vaccine (*n* = 599)	
	OR (95% CI)	*P*	OR (95% CI)	*P*
Female vs male	1.02 (0.69–1.50)	0.9	0.55 (0.37–0.83)	0.004
Age (per 10 years)	1.03 (0.88–1.20)	0.7	1.28 (1.07–1.54)	0.007
Parents from endemic region^a^	2.42 (1.57–3.74)	<0.001	4.17 (2.74–6.34)	<0.001
Travelled to endemic region^a^	2.42 (1.57–3.74)	<0.001	4.53 (2.95–6.93)	<0.001
Received care in endemic region^a^	2.95 (1.80–4.84)	<0.001	5.99 (3.59–10.00)	<0.001
Transfusion before 1992	1.86 (0.54–6.46)	0.3	1.24 (0.48–3.21)	0.7
Acupuncture	0.70 (0.41–1.20)	0.2	1.03 (0.62–1.73)	0.9
Tattoos	0.78 (0.47–1.30)	0.3	1.50 (0.91–2.46)	0.11
Piercing	1.17 (0.80–1.72)	0.4	0.84 (0.57–1.24)	0.4
Close contact with HBV + individual	1.18 (0.61–2.26)	0.6	1.15 (0.65–2.02)	0.6
Men who have sex with men	1.33 (0.77–2.31)	0.3	1.18 (0.75–1.84)	0.5
Nasal drug-use	0.67 (0.40–1.13)	0.14	0.69 (0.40–1.18)	0.18
Intravenous drug-use	0.35 (0.04–3.35)	0.4	2.71 (0.80–9.14)	0.11
Long-term stay at a medical centre	1.75 (0.71–4.31)	0.2	1.13 (0.39–3.21)	0.8
Previously incarcerated	0.62 (0.29–1.35)	0.2	2.99 (1.17–7.60)	0.02
HBV-prevalence of birth region				
Low (<2.0%)	1.00		1.00	
Intermediate (2.0–8.0%)	2.51 (1.54–4.08)	<0.001	7.01 (4.18–11.75)	<0.001
High (>8.0%)	0.51 (0.29–0.89)	0.02	2.86 (1.75–4.68)	<0.001
Health insurance plan				
Social security	1.00		1.00	
CMU^b^	2.69 (1.01–7.15)	0.048	1.46 (0.57–3.77)	0.4
AME^c^	1.46 (0.65–3.32)	0.4	2.30 (0.55–9.51)	0.3
Other	1.15 (0.16–8.26)	0.9	^d^	
None	1.37 (0.68–2.75)	0.4	6.02 (2.60–13.93)	<0.001
Nb of life-time sexual partners				
0–1	1.00		1.00	
2–9	0.40 (0.18–0.89)	0.03	1.36 (0.55–3.39)	0.5
⩾10	0.34 (0.16–0.75)	0.007	0.84 (0.33–2.09)	0.7
>1 sexual partner within 12 mo.s	1.13 (0.77–1.66)	0.5	0.84 (0.54–1.31)	0.4

Data from the Optiscreen-B study conducted from September 2010 to August 2011 in Paris, France among individuals reporting having been tested for hepatitis B virus (HBV). Discordant defined as a self-report HBV-status that did not correspond to the participant's serological test.

a Endemic defined as a region with moderate or high HBV prevalence. Travel period must have been longer than 3 months.

b *Couverture médicale universelle*, health insurance coverage that is given to persons living in precarious situations (i.e. unemployed, poverty, etc.).

c *Aide médicale d’état*, health insurance generally given to immigrants without proper documentation.

d Parameter estimates were unable to be obtained.

Of the 599 participants with self-reported history indicating effective vaccine, 446 (74.5%) were verified to have been vaccinated (with median anti-HBs antibody level at 459 mIU/ml, IQR = 90->1000) and 153 (25.5%) had a discordant serological result. Of the latter group, 100 (65.4%) were non-immunised, 50 (32.7%) had resolved infections (with median anti-HBs antibody level at 344 mIU/ml, IQR = 60->1000) and three (2.0%) had HBsAg-positive serology. Determinants for discordance in vaccinated self-reported history were male gender (*P* = 0.004); increased age (*P* = 0.007); having parents from, travelling to, receiving care in, or originating from a region of intermediate/high HBV-prevalence (*P* < 0.001); lacking any health insurance plan (*P* < 0.001); and previously incarcerated individuals (*P* = 0.02) (Table 4). Again, no multivariable analysis was conducted due to strong collinearity between variables.

Discordance for individuals declaring past infection was not common (16.4%), as was for the few participants declaring chronic infection (30.0%) (Table 3). The risk-factor analysis was precluded by the small patient sizes and low discordances in these self-report groups.

## Discussion

In this study conducted in the Paris metropolitan region among mostly MSM and immigrants, we observed that almost one-third of participants did not have a serological HBV-status corresponding to their past clinical description. Self-reporting is known to be an inaccurate reflection of vaccinated [14–17, 20, 21] and susceptible [14, 17, 22, 23] at-risk individuals. By asking questions on both vaccination and HBV testing practices, we build on these previous reports to encompass a slightly more extensive evaluation of self-reported HBV-disease status. These findings bear particular importance for screening campaigns aimed at individuals belonging to various HBV-transmission risk groups who have been previously tested.

With an estimated 38 HBsAg tests per 1000 habitants and a 0.8% HBsAg-positive seroprevalence [24], HBV-testing could be viewed as redundant for many individuals in France and perhaps other screening methods could reduce this inefficiency. One potential option could be determining HBV-status from previous patient information, while hypothetically, any person correctly identifying their status through self-reports would not need serological confirmation. The proportion of discordant results could then be viewed as the proportion of individuals in need of further testing. Data from our study would suggest that roughly half of the patients declaring negative HBV-status and a quarter of patients with other self-reported status would benefit from an HBV serological test. Improvement in reducing unnecessary tests could also be achieved by including information from patient medical charts [25], yet this might not be feasible for many at-risk groups, such as immigrant populations seeking care for the first time.

The group with the highest discordance was individuals with negative self-reported status, with most discordant participants having serological evidence of vaccination. In parallel, most discordant participants describing effective vaccine from their clinical history were in fact non-immunised. From the risk-factor analysis, the groups with discordant negative and vaccinated HBV-status were similar: mainly individuals linked to regions of intermediate/high HBV-prevalence and not under the national health insurance plan. Knowledge about HBV transmission factors are generally good in these patient groups compared with others [26], yet they have evidenced difficulties in differentiating between negative and immunised statuses [27]. Moreover, general practitioners in France sometimes have difficulty in correctly interpreting HBV serologic results [28], possibly leading to further confusion among these individuals. More fostered education on HBV-status by trained specialists has demonstrated success in increasing understanding of HBV disease in Hmong and Asian/Pacific Islanders in the USA [29, 30] and could be of use in this setting.

Other hypotheses could account for why individuals claiming to be vaccinated were, in fact, non-immunised or had resolved the infection. First, anti-HBs antibodies are known to wane many years after vaccination for most individuals [31] and those without antibodies could have indeed been vaccinated at a younger age but lost antibodies at the time of participating in the study. It might explain why higher age was significantly associated with discordant ‘effective vaccine’ status. However, almost 90% reported receiving the vaccination during adolescence or adulthood and in light of the antibody levels detected in this group, the last vaccine booster could have been administered within the past decade [32]. Second, inadequate vaccine response is observed in 5% of immunocompetent individuals and is more frequent in other populations (i.e. older individuals, renal insufficiency, immunocompromised, etc.) [33]. Individuals could have in fact been vaccinated but failed to achieve an appropriate response. Finally, 8% of individuals claimed to be vaccinated, yet in fact, had resolved infection. These individuals could have had resolved infection prior to vaccination, while either no pre-vaccine screening had taken place or only anti-HBs and not anti-HBc antibodies were tested. Vaccination might not be effective for this patient profile [34].

Other studies have shown that self-reports of vaccine history are poor indicators of true immunised status in mostly IDUs, MSM and younger populations seeking STD screening, with predictive sensitivities ranging from 24 to 55% [14, 16, 17]. In contrast, we observed a much higher sensitivity, with an overall 86% correctly identifying their vaccinated status, which was even higher in the at-risk groups mentioned above (i.e. IDUs, MSM). The reasons for these discrepancies are difficult to explain. Compared with others, our study population had a much higher representation of lower risk groups with regular healthcare, for whom HBV-vaccination declaration could be more accurate. However, the research supporting this claim, wherein the sensitivities of vaccinated self-reports are compared between various risk-factors groups, is almost non-existent (apart from the one report demonstrating no difference in sensitivity among IDUs *vs.* non-IDUs [21]).

Importantly, the only discordance of participants reporting chronic infection was among those who in fact had resolved infection, whereas the most frequent serological status of discordant participants reporting past infection had HBsAg-positive serology. HBsAg-negative participants assuming to have a chronic infection would have received unnecessary follow-up with a negligible risk of liver-related morbidity and thus the clinical ramifications would be considered minimal. In contrast, HBsAg-positive participants assuming to have past infection, even negative infection or vaccinated status, could lack appropriate care, progress to later stages of disease without treatment and continue transmitting HBV to others. Testing or re-testing these individuals, however, does not guarantee that they will be linked-to-care [35, 36], regardless of patient-oriented assistance [37] and the care received might not be fully adequate for viral hepatitis [38]. Taken together, these observations support the need for improved quality in the continuum-of-care for chronic hepatitis B.

For other discordant groups, the seriousness of their clinical outcomes in the absence of serological confirmation is debatable. Vaccinated participants whose self-reported clinical history coincides with a negative or past infection would require no further follow-up, except for possibly serological confirmation of protective immunity. Past-infected patients in whom negative or effective vaccination was reported might lack HBV-appropriate care. Risk of liver morbidity and mortality is exceedingly low in this patient group [39]; however, reactivation can occur under certain circumstances and might complicate clinical follow-up while undergoing anti-hepatitis C virus or immunosuppressive therapies [40]. Finally, any HBsAg-negative/anti-HBs antibody-negative individual reporting effective vaccination would unknowingly remain susceptible to infection. Vaccination after testing these individuals is paramount, yet uncommon in large campaigns such as ours [19].

Certain limitations of our study need to be addressed. First, we neither collected the date of past test nor did we ask questions on at-risk HBV activities since the last test. It was then difficult to assess whether participants with self-reported negative status had discordance due to changing from negative to positive status during this period. Second, the study population only reflected individuals who were able to recall their HBV-test result. Including individuals unsure of their HBV-status or even without a previous test would evidently modify discordance results; however, they would require testing regardless of disease status based on self-reported patient history and thus irrelevant to the study question. Third, individuals were only asked if they had been vaccinated and not whether they had received enough vaccinations to provide adequate protection, which is implied by the definition of ‘effective vaccine.’ We did ask participants the number of vaccines received and one-fifth of those with available data reported only one vaccine. Attempts were made to include this information when defining self-reported HBV status, yet these did not increase classification agreement or concordance (data not shown). Finally, we did ask the date at which vaccination occurred. As most participants only responded in categories of ‘at birth, adolescence, or adulthood’; the time since vaccination was difficult to fully assess.

In conclusion, HBV-status based on self-reported accounts of vaccination and previous testing is poor in confirming uninfected and to a lesser extent, vaccinated and infected individuals in this large screening study. These findings stress the need for serological testing to confirm and reinforce self-reports among at-risk individuals. For clinicians and public health workers, appropriately explaining HBV-status to individuals after testing bares considerable importance, especially for those from moderate to high HBV-endemic countries, in order to ensure appropriate care.

## Supplementary material

For supplementary material accompanying this paper visit https://doi.org/10.1017/S0950268818002650. click here to view supplementary material
