# Supplementary material for: Self-reported patient history to assess hepatitis B virus serological status during a large screening campaign
Source: Epidemiol Infect. 2018 Sep 28;147:e16. doi: 10.1017/S0950268818002650 (PMC6518477; doi:10.1017/S0950268818002650)
Supplement: Supplementary file 1 [file S0950268818002650sup001.docx]

**SUPPLEMENTARY MATERIAL**

**Supplementary Table 1. Comparison of individuals included versus non-included in analysis**

|  | | Included | Not included | *p*^§^ |
| --- | --- | --- | --- | --- |
|  | | (*n*=1090) | (*n*=2907) |  |
| Male | | 647 (59.4) | 1591 (54.7) | 0.009 |
| Age^‡^ [N=3996] | | 32 (25-42) | 33 (25-47) | <0.001 |
| HBV prevalence of birth country | |  |  |  |
|  | Low (<2.0%) | 694 (63.7) | 1530 (52.6) |  |
|  | Intermediate (2.0-8.0%) | 224 (20.6) | 716 (24.6) |  |
|  | High (>8.0%) | 172 (15.8) | 661 (22.7) |  |
| Parents born in high HBV-endemic region [N=3984] | | 293 (27.0) | 846 (29.2) | 0.16 |
| Travelled to high HBV-endemic region^¶^ | | 286 (26.2) | 897 (30.9) | 0.004 |
| Sought care in high HBV-endemic region | | 202 (18.5) | 620 (21.3) | 0.05 |
| Health insurance plan | |  |  | <0.001 |
|  | Social security | 919 (84.3) | 2123 (73.0) |  |
|  | CMU^††^ | 45 (4.1) | 184 (6.3) |  |
|  | AME^‡‡^ | 40 (3.7) | 141 (4.9) |  |
|  | Other | 6 (0.6) | 23 (0.8) |  |
|  | None | 80 (7.3) | 436 (15.0) |  |
| Received transfusion before 1992 | | 37 (3.4) | 99 (3.4) | 0.9 |
| Received acupuncture | | 169 (15.5) | 372 (12.8) | 0.03 |
| Received tattoos | | 174 (16.0) | 368 (12.7) | 0.007 |
| Received piercing | | 488 (44.8) | 1247 (42.9) | 0.3 |
| Close contact with an HBV+ individual | | 122 (11.2) | 187 (6.4) | <0.001 |
| Number of life-time sexual partners | |  |  | <0.001 |
|  | 0-1 | 75 (6.9) | 636 (21.9) |  |
|  | 2-9 | 428 (39.3) | 1318 (45.3) |  |
|  | ≥10 | 587 (53.9) | 953 (32.8) |  |
| >1 sexual partner within the last 12 months | | 650 (59.6) | 1164 (40.0) | <0.001 |
| Men who have sex with men | | 247 (22.7) | 172 (5.9) | <0.001 |
| Nasal drug-use | | 180 (16.5) | 256 (8.8) | <0.001 |
| Intravenous drug-use | | 16 (1.5) | 7 (0.2) | <0.001 |
| Long-term stay at a medical centre | | 43 (3.9) | 117 (4.0) | <0.001 |
| Previously incarcerated | | 59 (5.4) | 148 (5.1) | 0.7 |

Data from the Optiscreen-B study conducted from September 2010-August 2011 in Paris, France.

^‡^Median (IQR) given.

^§^Comparisons between inclusion groups were performed using Kruskal-Wallis test for continuous variables and Pearson χ² test for categorical variables.

^¶^Period of stay was longer than 3 months.

^††^*Couverture médicale universelle*, health insurance coverage that is given to persons living in precarious situations (i.e. unemployed, poverty, etc.)

^‡‡^*Aide médicale d’état*, health insurance generally given to immigrants without proper documentation.

**Supplementary Table 2. Self-reported HBV-infection status (based on previous HBV test alone) compared to serological results**

| Serological HBV-status** | Previous HBV-test* | | | |
| --- | --- | --- | --- | --- |
|  | Negative | Effective vaccine | Past infection | Chronic infection |
|  | (*n*=835) | (*n*=181) | (*n*=59) | (*n*=15) |
| Non-immunized | 305 (36.5) | 13 (7.2) | 2 (3.4) | 0 (0) |
| Vaccinated | 417 (49.9) | 156 (86.2) | 3 (5.1) | 0 (0) |
| Resolved infection | 106 (12.7) | 12 (6.6) | 49 (83.1) | 6 (40.0) |
| HBsAg-positive | 7 (0.8) | 0 | 5 (8.5) | 9 (60.0) |

Agreement=47.6%, unweighted κ=0.23

*Based on response to following question: ‘What was the result of [your last] HBV test?’

**Disease status was defined according to serological results (given in Box 1).
